# A simple method for microwave-assisted preparation of tire samples

**DOI:** 10.1038/s41598-023-47309-z

**Published:** 2023-11-18

**Authors:** Renchao Zhu, Yingqi Yuan, Yu Yang, Qiyue Yang, Aihua Yu

**Affiliations:** College of Civil Engineering, Nanjing Forestry University, No. 159 Longpan Road, Nanjing, 210037 People’s Republic of China

**Keywords:** Environmental impact, Characterization and analytical techniques, Environmental sciences, Materials science

## Abstract

Heavy metals content in tires affects the safety of soil and agricultural products. The digestion method is a pretreatment for determining heavy metals in tire samples, and will affect the efficiency and accuracy of the heavy metal determination. The microwave digestion process and reagents for tire samples are not currently standardized. Therefore, this study attempts to provide an appropriate method of resolution for scholars. All digestion processes were performed in Mars One. We tested 15 different acid mixtures to determine the best reagent type and dose and then investigated the effect of maximum temperature, holding time, and sample grams on the degree of digestion. In summary, the best condition to digest the tire sample was a mixture of 3 ml HNO_3_ and 7 ml H_2_SO_4_, taking 0.1 (± 0.0005) g tire sample, at the maximum digestion temperature of 220 °C for 25 min. The experimental conclusion will provide a reliable experimental method for scientists using MARS One to study heavy metals in tires. At the same time, researchers using the MARS series can also find valuable references in this paper.

## Introduction

In many countries, heavy metals pollution has become a significant concern. Due to their toxicity, persistence, and bioaccumulation (in plants, animals, soil, water, and sediments)^1^, heavy metals have been identified as harmful environmental pollutants^2–4^. They spread to the surrounding soil through road runoff, atmospheric deposition, and other means, so heavy metals content in roadside soils is well above their background content^5–7^. Roadside plants and crops such as Chinese cabbage^8^, tomato, red pepper^9^, wheat, and rice^10^ are also directly or indirectly affected, absorbing heavy metals from the soil via their foliage and roots^11^. Consumption of contaminated food can adversely affect human health, especially in children^12–14^.

**Figure 1:**
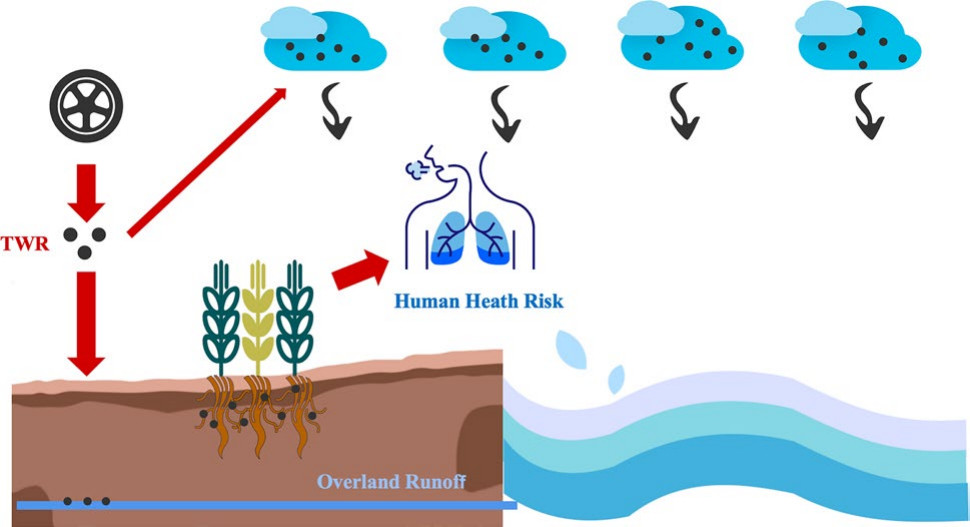
The contamination of tires.

Natural factors (e.g., weathering of rocks, volcanic eruptions, soil formation processes, and forest fires) and human activities (e.g., industrial emissions, fuel combustion, waste incineration, transport, and agricultural activities) are responsible for the accumulation of heavy metals in the environment^4,15^. Among them, the human factor is the most significant^16^. Traffic emissions are a primary source of heavy metals in roadside soils and crops^17–19^, and tire wear is an essential component of traffic emissions^20,21^. Tire wear particles (TWP) emissions account for 5–30% of non-exhaust emissions from transport. The mass of TWP generated is estimated to be 1,327,000 t/a for the European Union, 1,120,000 t/a for the United States and 133,000 t/a for Germany^22^. TWP emissions are projected to increase steadily over the next decade^23^. The contamination of the tire is shown in Fig. 1.

**Figure 2:**
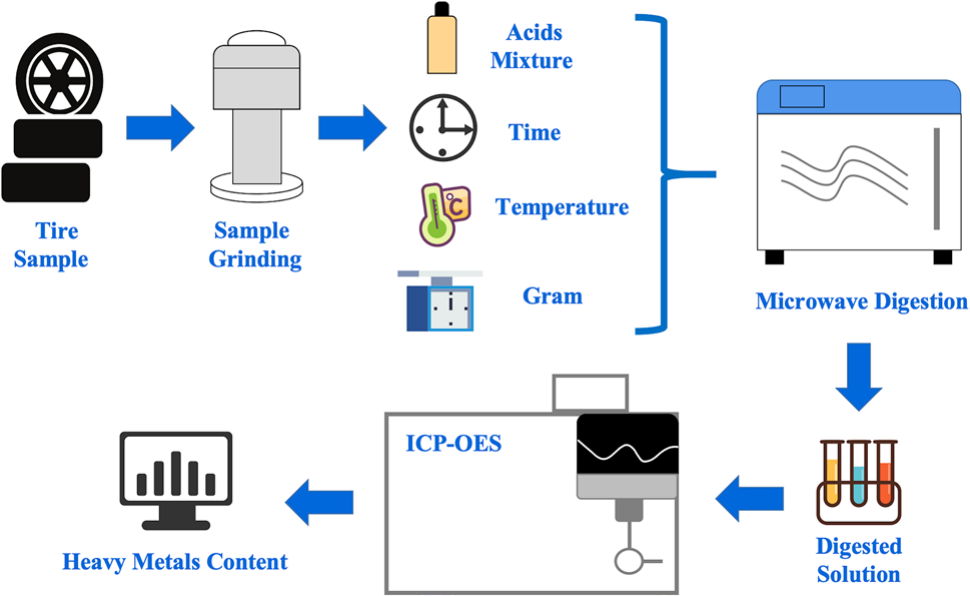
The completion process for the determination of heavy metals in tires.

Tire rubber is a common component of municipal solid waste (MSW)^24^. Over the lifetime of a tire, approximately 30% of its tread material is released to the environment as TWP^25^. Approximately 50% of the TWP can be expected to remain in the roadside soil, while others are likely to reach the aquatic environment through run-off and atmospheric transport^26,27^. Hence, the continued accumulation of TWP may eventually cause widespread environmental health problems^28^. Tire wear and tire corrosion can release many trace metals, such as cadmium (Cd), cobalt (Co), chromium (Cr), copper (Cu), mercury (Hg), manganese (Mn), molybdenum (Mo), tungsten (W), nickel (Ni), and lead (Pb)^21,29^. As a result, tires are predicted to be the major anthropogenic source of roadside zinc (Zn) in the atmosphere, about four times greater than brake wear and greater than other potential sources such as galvanised street furniture, car bodies and engine oil^30,31^. Tire rubber, either stored as end-of-life tires or recycled into rubber products, has been linked to the release of heavy metals into the environment, in addition to wear and tear^23^. Therefore, in addition to studying the recycling^32^ and reuse^33^ of tires, determining and analysing the heavy metals content of tires are critical to monitoring and quantifying the environmental contribution of TWP emissions^34,35^. The completion process for the determination of heavy metals in tires is shown in Fig. 2.

However, sample preparation is the most critical and time-consuming step in the analysis process, taking up almost two-thirds of the total analysis time^36^. Traditional digestion methods include wet digestion and dry ashing, which are time-consuming and require many operators' attention, skill, and experience^37^. Unlike conventional methods, microwave digestion significantly reduces digestion time (2–5 times) and has other advantages, such as reduced contamination, reagent, sample consumption, loss of volatiles, and improved safety^38^. Quite a few scientists have researched microwave digestion. Kuss^39^ cited literature before 1992 on the application of microwave digestion in elemental analysis, Zlotorzyns^40^ discussed the fundamentals of microwave interaction with the sample matrix, and De^41^ introduced a microwave-assisted technique for the determination of heavy metals in sewage sludge. Smith^42^ reviewed the application of microwave-assisted sample preparation in analytical chemistry. Microwave-assisted sample preparation is widely used in experiments to convert solid samples into representative solutions that spectrochemical methods can quickly analyse, such as inductively coupled plasma optical emission spectrometry (ICP‒OES) or inductively coupled plasma‒mass spectrometry (ICP‒MS)^32^. In addition, microwaves are also used in many polymer processing technologies: the surface treatment process of superabsorbent polymers (SAPs) based on poly (sodium acrylate)^44^ and the structural changes of potassium permanganate-oxidized polyacrylonitrile-based fibers^45^.

Carbon black, an excellent reinforcing filler in tires, gives tires good tensile strength, tear resistance, and abrasion resistance^46^. It is also a challenge to use traditional techniques to dissolve this material. The process of digestion is a difficult task^47^. Therefore, the microwave digestion process and reagents for tire samples are not currently standardized. Therefore, this study aimed to develop a microwave digestion method suitable for the routine preparation of tire samples for heavy metals analysis according to the recommendations of the CEM Mars One Manual.

This study investigates the effects of reagent type, dose, temperature, time, sample quality, and others on the degree of digestion to find the most effective combination. We hope to provide a reliable experimental method for scholars who use MARS One to study heavy metals in tires and provide a valuable reference for scholars who use MARS series products.

## Materials and methods

### Instrumentation

Tire digestion experiments were conducted in a benchtop microwave digestion system (CEM Mars one, manufactured by CEM Corp., USA) with a maximum power of 1000 W and a temperature control system to detect and control the temperature conditions in the sample container. The turntable supplied by CEM can hold up to 16 digestion vessels. The vessel body and gasket are made of polytetrafluoroethylene (PTFE), and the lid is made of polyflouroalkoxy (PFA) with a 3.2 mm diameter vent in the centre of the lid to relieve pressure and minimize acid loss.

### Reagents and Samples

All reagents were of analytical grade and 99% pure. Nitric acid (HNO_3_ 65–68%, China), hydrogen peroxide (H_2_O_2_ 30%, China), hydrochloric acid (HCl 36–38%, China), hydrofluoric acid (HF ≧40%, China), and sulfuric acid (H_2_SO_4_ 95–98%, China) were used for sample digestion. Deionized water (China) was used for dilution, so laboratory utensils, digestion vessels, etc., were thoroughly cleaned and then continuously immersed in 10% HNO_3_ solution after use. Ultrapure water (China) was used for the constant volume of digestion.

We selected the most representative Michelin tire (France) as samples. Referring to "GB/T 15340-2008 Rubber, raw natural and raw synthetic-Sampling and further preparation procedures^48^", we randomly took 10 5 cm × 5 cm small pieces from different parts of the tire (side, tread) in a car repair shop. All samples were ground and sieved through a 100-mesh nylon screen (China), and 500 g were taken as a sample after mixing.

### Quality control

The reagents and chemicals used were of analytical grade with a purity of 99%. To minimize the risk of contamination, all containers were soaked in 10% HNO_3_ (65–68%) for 12 h, rinsed three times with deionized water, and dried in an oven at 60–65 °C for 24 h. The test was repeated three times for each sample.

After grinding and sieving, the sample weighed (0.1 ± 0.0005) grams (g), was accurately weighed, and the sample quality relative error was not more than 0.001 g; that is, the close error of sample quality was not more than 0.5%. In this way, we were able to ignore the effect of the weighing error.

A total of 0.1–0.5 g of solid sample and 5–10 ml of reagent were added, using an even number of vessels for each digestion. To avoid explosion and other hazards, we set the maximum temperature of the instrument below 230 °C. In addition, after each digestion, the digestion tank could only be removed if the temperature was below 80 °C within 20 min.

### Experimental method

Generally, the digestion of tires is done by microwave digestion method, but the digestion effect of different types of microwave digestion instrument and different digestion reagents is different. It is widely accepted that a complete digestion is yellowish-white or clear and free of solid residues^49–53^. We determined the best method of microwave digestion of tire by changing the parameter conditions of microwave digestion each time, including reagent type, reagent dose, the digestion procedure (temperature, time), and sample gram number.

## Results and discussion

### Influence on microwave digestion of different acid systems

After grinding and sieving, we placed 0.1 ± 0.0005 g tire samples in the digestion vessels. Then we added different combinations of five acids in the digestion vessels to investigate the influence of different types of acids on the degree of microwave digestion. Following the instrument's instruction manual, we set the initial digestion procedure as shown in Table 1.

**Table 1:** Microwave-assisted heating program.

Step	Warming time (min)	Holding time (min)	Temperature (°C)	Applied power (W)
1	10:00	5:00	160	900
2	10:00	20:00	200	900

**Figure 3:**
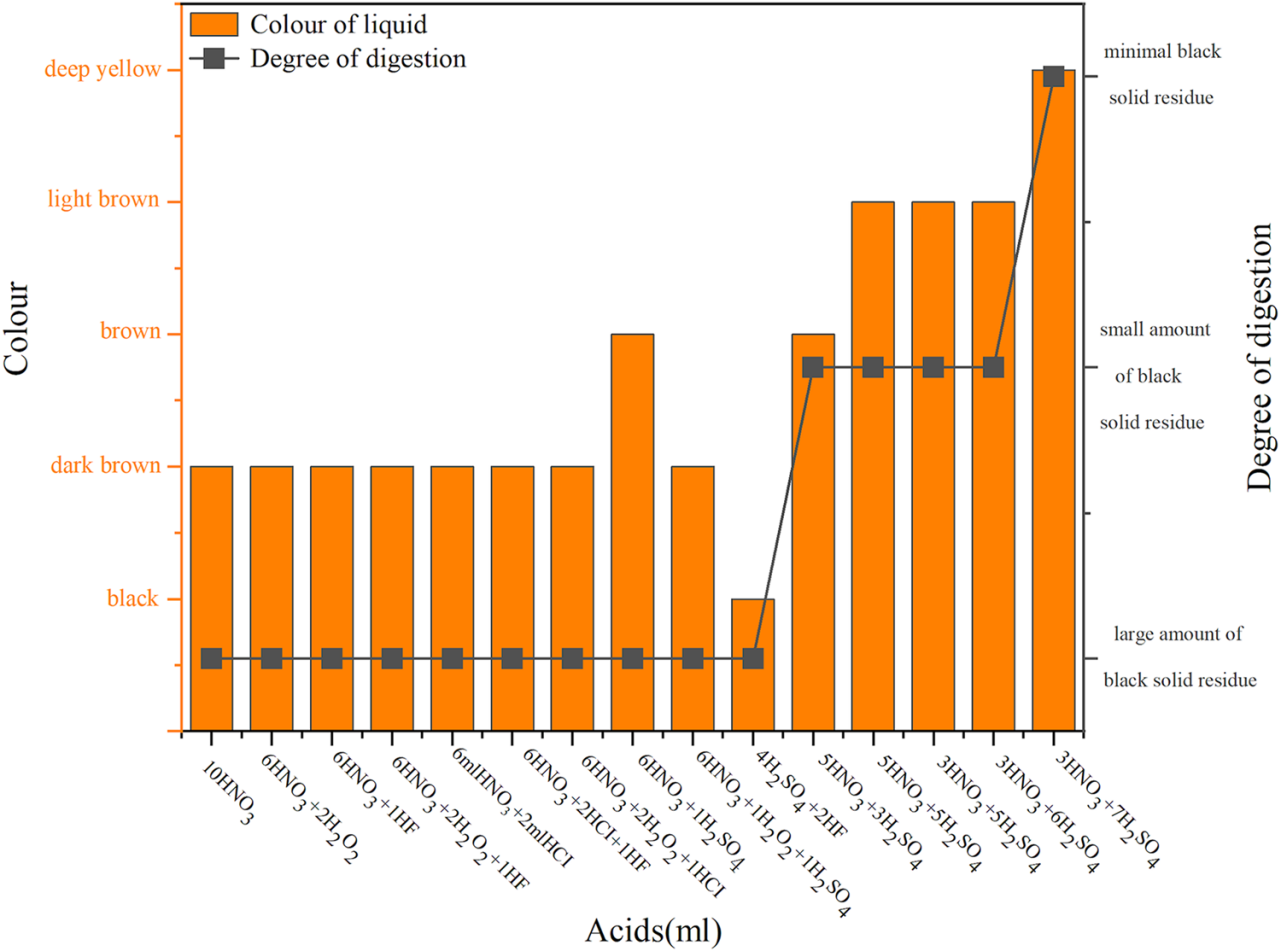
The effect of digestion with different combinations of acids.

The mixture of HNO_3_ and H_2_SO_4_ had a good digestion effect on the tire samples (Fig. 3). Using a mixture of 6 ml HNO_3_ and 1 ml H_2_SO_4_ and a mixture of 5 ml HNO_3_ and 3 ml H_2_SO_4_ changed the colour of the liquid from dark to brown. Furthermore, using the mixture of 5 ml HNO_3_ and 3 ml H_2_SO_4_ left only a small amount of black residue in the digestion solution. Therefore, we adjusted the dosages of HNO_3_ and H_2_SO_4_ to explore the best acid system. It is worth noting that when the mixture of HNO_3_ and H_2_SO_4_ was added to the digestion vessel, an exothermic reaction occurred. Therefore, we needed to place the digestion vessel in a fume cupboard for half an hour to achieve the role of predigestion.

**Figure 4:**
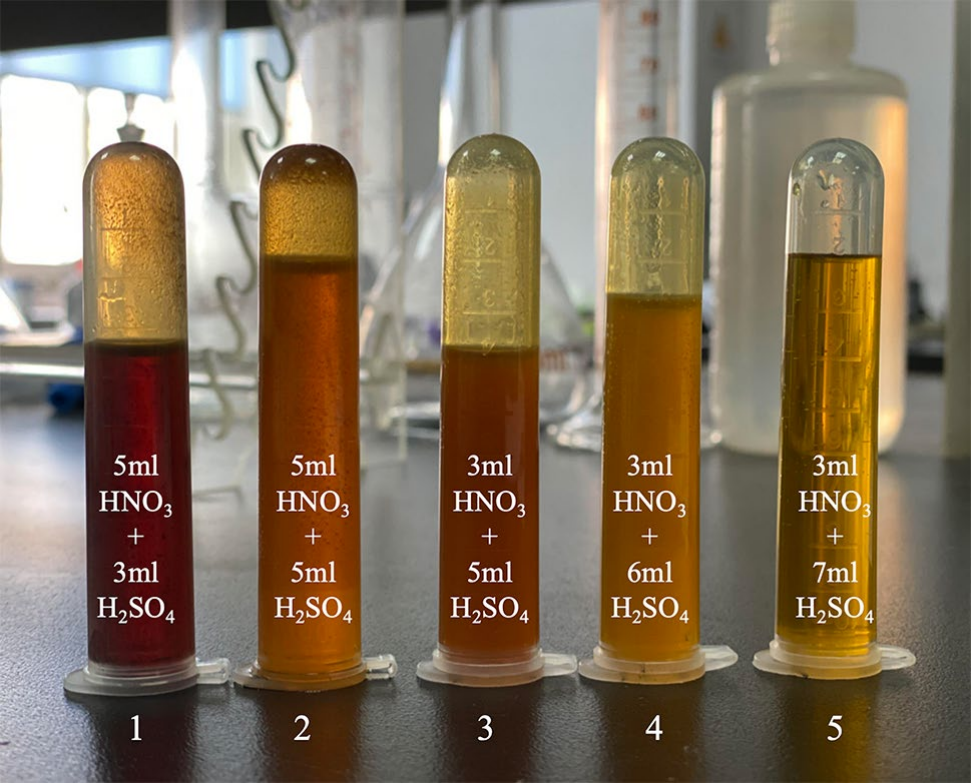
The digestion effect of HNO_3_-H_2_SO_4_.

As shown in Figs. 3 and 4, the degree of digestion improved, as the consumption of HNO_3_ decreased and the consumption of H_2_SO_4_ increased. The mixture of 3 ml HNO_3_ and 7 ml H_2_SO_4_ had almost completely dissolved the tire, with only a small solid residue, which was light yellow after a constant volume of 25 ml through ultrapure water. Having determined the best combination of acids, we researched the influence of digestion temperature and holding time on digestion to optimize the digestion scheme further.

### Influence of temperature and time on the microwave digestion rate

Polymer digestion processes reach high temperatures, and combustion is a very efficient way of destroying matrices (including organic additives)^47,54^. Therefore, the complete dissolution of the tire sample often depends on the highest temperature and holding time during digestion.

#### Influence on microwave digestion of maximum temperature

Step 1 remains the same, and the changes in Step 2 are shown in Table 2. After grinding and sieving, we added 0.1(± 0.0005) g tire samples and a mixture of acids in digestion vessels. After predigestion, the vessel lid was tightened and placed in the microwave digestion apparatus, and different maximum temperatures were set for digestion.

**Table 2:** Maximum temperature variation of step 2.

Serial number	Warming time (min)	Holding time (min)	Maximum temperature (°C)	Applied power (W)
1	10:00	20:00	180	900
2	10:00	20:00	190	900
3	10:00	20:00	200	900
4	10:00	20:00	210	900
5	10:00	20:00	220	900

Too low a temperature affects the degree of digestion, while too high a temperature increases the cooling time and the pressure inside the instrument, increasing the risk. Therefore, we set the maximum temperature to between 180 and 220 °C.

**Figure 5:**
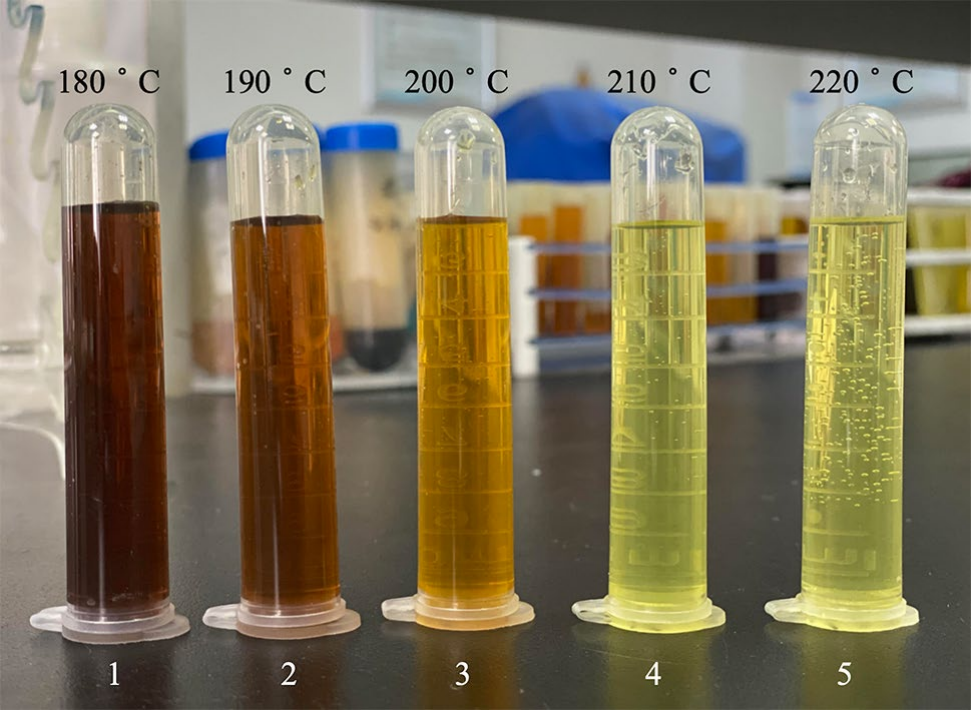
The effect of digestion with different maximum temperatures.

As shown in Fig. 5, when the temperature was below 200 °C, there was still a small amount of solid residue in the digestion solution after digestion, and the tire was not fully digested. As the temperature rose, the effect of digestion improved. When the temperature reached 210 °C and 220 °C, the tire was completely digested, and the liquid had no solid residue. The liquid was colourless and transparent when diluted to 25 ml with ultrapure water. The higher the temperature, the higher the degree of digestion, so we chose 220 °C as the best temperature for tire digestion in a safe and stable experiment.

#### Influence of holding time on microwave digestion

The holding times in Table 3 were optimized to maximize efficiency under the assumption of complete digestion, as the comprehensive digestion program is mainly influenced by the retention time at the highest digestion temperature. As shown in Table 4, digestion was carried out by setting different holding times.

**Table 3:** Microwave-assisted heating program.

Step	Warming time (min)	Holding time (min)	Temperature (°C)	Applied power (W)
1	10:00	5:00	160	900
2	10:00	20:00	220	900

**Table 4:** The effect of digestion with different holding times.

Serial number	Maximum temperature (°C)	Holding time (min)	Degree of digestion	Colour of digestion solution
1	220	10:00	Minimal solid residue	Yellow
2	220	15:00	No solid residue	Light green
3	220	20:00	No solid residue	
4	220	25:00	No solid residue	Nearly colourless
5	220	30:00	No solid residue	

**Figure 6:**
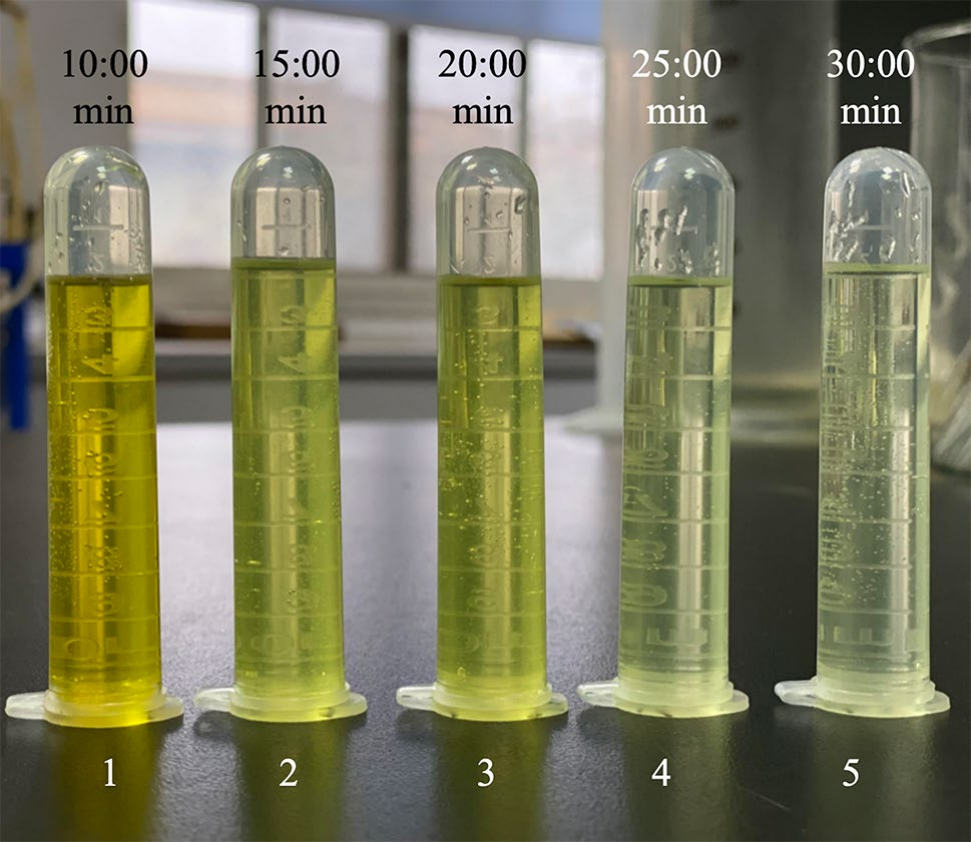
The effect of digestion with different holding times.

In Fig. 6, under the effect of high temperature, the tire sample was almost completely dissolved even if held for only 10 min. When the holding time reached 25 or 30 min, the liquid appeared virtually colourless and transparent, with the highest degree of digestion. After the digestion solution was diluted to 25 ml, it was still colourless and transparent. Therefore, we chose 25 min as the maximum temperature holding time to achieve the highest resolution in the shortest time.

**Figure 7:**
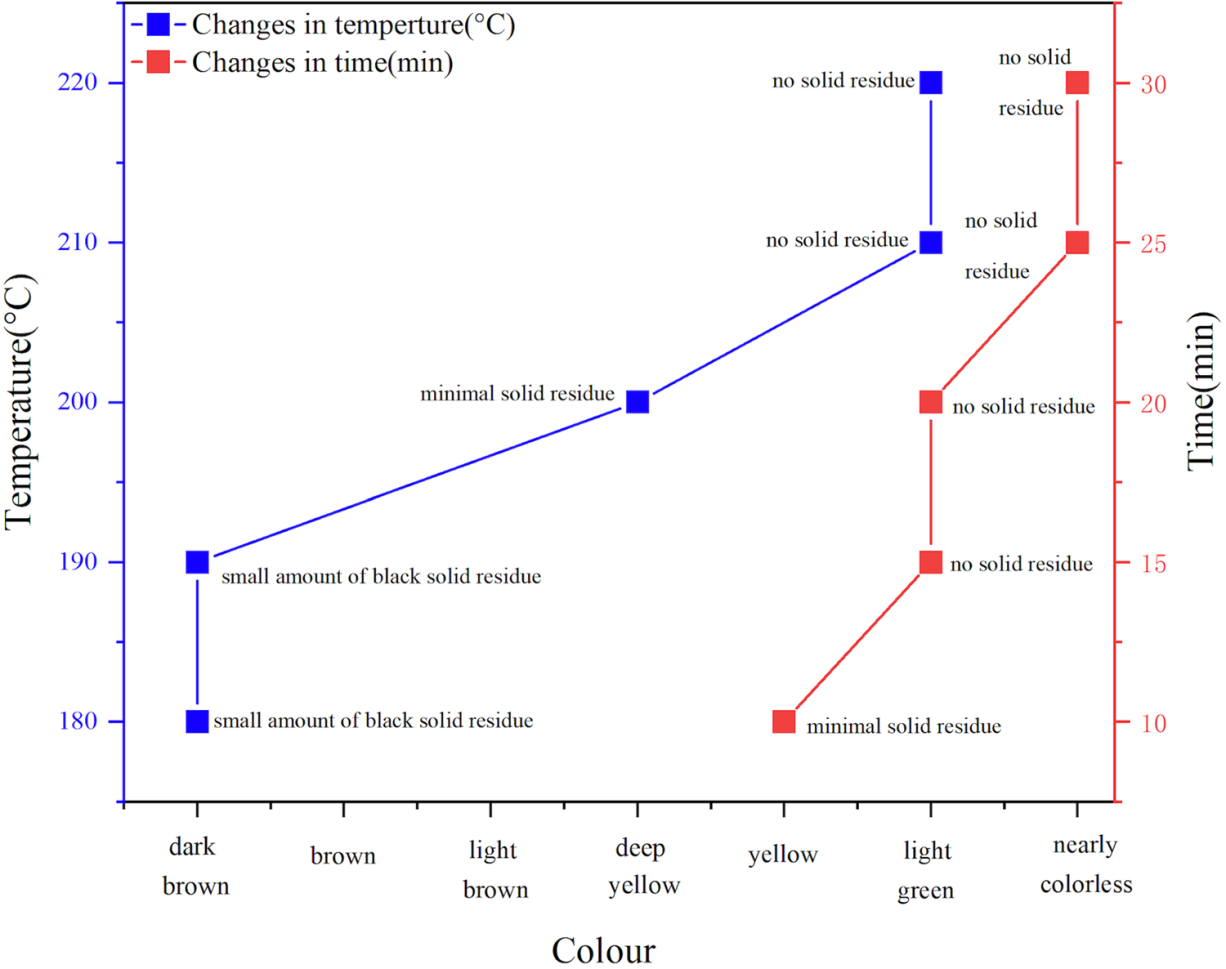
The effect of digestion with different temperatures and times.

From the result in Fig. 7, the two factors separate into two groups: (a) higher temperatures result in higher digestibility and clearer liquid and (b) longer holding time, higher digestion level, clear liquid. Therefore, we determined out the best microwave- assisted heating program (Table 5).

**Table 5:** Microwave-assisted heating program.

Step	Warming time (min)	Holding time (min)	Temperature (°C)	Applied power (W)
1	10:00	5:00	160	900
2	10:00	25:00	220	900

### Influence on microwave digestion of grams of sample

Practical experience has shown that it is impossible to guarantee that each weighing is exactly 0.1 g, so the microwave digestion program must have some ability to resist the influence of sample mass variations. Moreover, the digestion process is also influenced to some extent by the grams of the tire sample. Therefore, we researched the influence of different grams of samples on digestion. The treated tire samples were weighed in grams and placed in digestion vessels. After adding acid, digestion was carried out according to the microwave-assisted heating program (Table 5). The results are shown in Table 6.

**Table 6:** The digestion effect of different grams of sample.

Serial number	Sample grams (g)	Degree of digestion	Colour of digestion solution
1	0.1 ± 0.0005	No solid residue	Nearly colourless
2	0.12 ± 0.0005	No solid residue	Light green
3	0.14 ± 0.0005	No solid residue	
4	0.16 ± 0.0005	Small amount of white solid residue	
5	0.18 ± 0.0005	Small amount of white solid residue	

**Figure 8:**
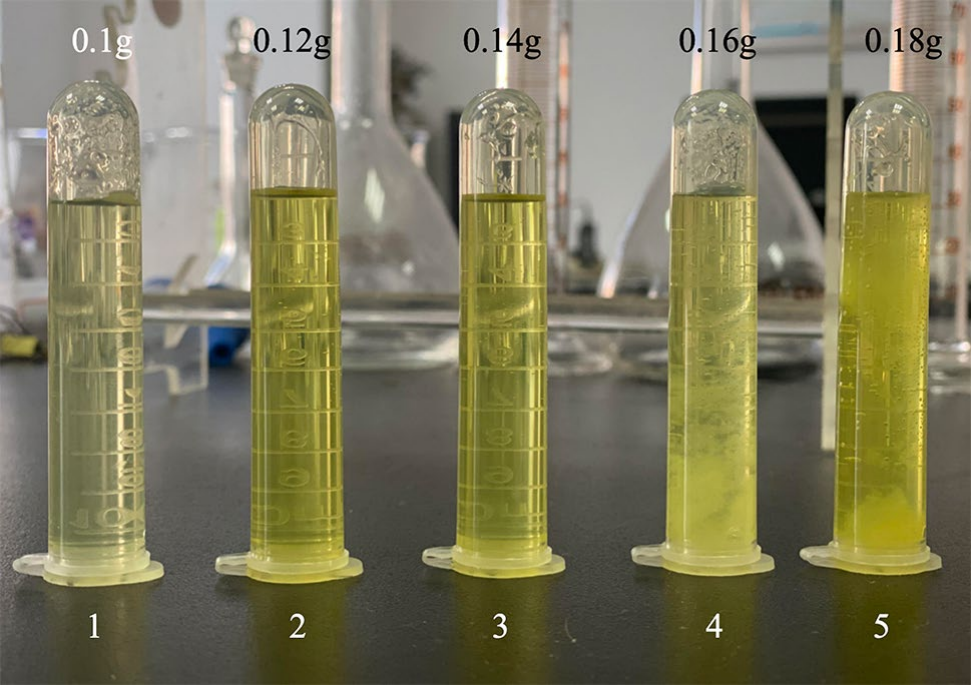
The effect of digestion with different grams of sample.

In Fig. 8, we found that samples could be wholly digested without residue when the gram was between 0.1 and 0.14 g. After diluting the digestion solution to 25 ml with ultrapure water, the solution was colourless and transparent. The effect of digestion was the best when the gram of sample was 0.1 g. However, when the gram increased to 0.16 g and 0.18 g, the sample could not be completely digested, and a small amount of white solid residue appeared in the digestion solution.

### Discussion

In the past, microwave digestion technology was often adopted for the pretreatment of animal, plant, and soil samples^55^. At present, researchers have proposed many microwave digestion schemes for some complex materials, such as spodumene, particulate matter (PM_2.5_), aquatic products, coke, and so on. The details are shown in the following Table 7. We can see that HNO_3_ is the most common acid in sample digestion, and it is a strong oxidizing agent to release elements in samples as soluble nitrates, and is well motivated by microwave^56^. Concentrated acids (HNO_3_, H_2_SO_4_, HCl and HF), mixed or not, are used in most complex sample digestion methods, increasing the efficiency of sample digestion^57^.

**Table 7:** Microwave digestion schemes for some complex materials.

Serial number	Sample	Acids	Holding time (min)	Temperature (°C)	References
1	Spodumene	H_2_SO_4_, phosphoric acids(H3PO4), HF	30	230	^43^
2	PM2.5	HNO_3_, HF, boracic acid(H3BO3)	–	200	^58^
3	Aquatic products	HNO_3_, H_2_O_2_	10	190	^59^
4	Coke	HNO_3_, HCl	55	260	^60^
5	Antifouling paints	HF, HNO_3_, H_2_O_2_	–	–	^57^
6	Sediment core	HNO_3_	20	200	^61^
7	Carbon nanotube	HNO_3_, and H_2_O_2_	35	270	^62^
8	Coal fly ash	HNO_3_, HCl, HF	25	190	^56^

Digestion results are closely related to acid type, temperature control, and other operational details^63^, especially for complex samples such as tires. Moraes studied the digestion effect of two acids mixtures based on holding them at 280 °C for 15 min (sample mass was 400 mg)^47^. The reagent volumes were: (i) 5 ml HNO_3_, 1 ml H_2_SO_4_, and (ii) 5 ml HNO_3_, 1 ml HCl and 1 ml H_2_O_2_. There are also a number of scientists involved in rubber tires research, as shown in Table 8. By analyzing all these methods, we concluded that HNO_3_ and H_2_SO_4_ positively affect the digestion of some complex samples. Furthermore, with almost all temperatures approaching 200 °C or above, the temperature seems to be the biggest factor that affects digestion. Neither of the two acids mixtures in Moraes' study was good at dissolving samples, but they were not investigated further. The sample mass of Nos. 1 and 2 in Table 8 is a range, the maximum temperature of No. 3 is also a range, No. 6 and No. 7 do not even give the grams of the sample, and No. 5 is no acid. In addition, the temperature of some methods is too high, which can pose safety risks. Therefore, we recognized that current rubber tires research is not comprehensive, and the microwave digestion process and reagents for tire samples are not currently standardized.

**Table 8:** Microwave digestion schemes for rubber tires.

Serial number	Gram (g)	Acids	Holding time (min)	Temperature (°C)	Instrument	References
1	0.005–0.05	9 ml HNO_3_, 1 ml H_2_O_2_	20	220	MARS 6 (CEM, Buckingham, UK)	^23^
2	0.1–0.23	5 mL HNO_3_, 3 mL deionized water	15	250	UltraCLAVE (Milestone, Italy)	^30^
3	0.4	6 mL HNO_3_, 2 mL H_2_O_2_	–	220–240	Multiwave, Rotor 8NXF100, (Anton Paar, Graz, Austria)	^31^
4	0.1	5 mL HNO_3_, 0.5 mL HF	20	200	MARS 5 (CEM Corporation, USA)	^67^
5	0.1	–	15	200	START D Microwave Digestion System (Milestone, Milan, Italy)	^68^
6	–	HNO_3_, HCl, H_2_O_2_	20	220	Milestone Ethos microwave	^68^
7	–	10 mL HNO_3_ and deionized water (ratio of 1:1)	10	200	Model ETHOS One, (Milestone, Inc.)	^64^
8	0.1 (± 0.0005)	3 ml HNO_3_, 7 ml H_2_SO_4_	25	220	CEM Mars one, (CEM Corp., USA)	This work

As a result, this study is a good complement to the research on rubber tires. We combined the study of complex samples and rubber tires and went through 15 acids mixtures to find the best one. At the same time, we refined the two factors of temperature and holding time to find the best solution. Moreover, compared to these methods, the method in this paper not only analyses the selection range of the gram of the sample but also avoids the use of dangerous and environmentally unfriendly HF, H_2_O_2_^65,66^.

## Conclusions

By changing the conditions of microwave digestion one by one, this study carried out much experimental work and finally determined the best scheme for microwave digestion of tire samples as follows:

- Take a 0.1 (± 0.0005) g tire sample.
- A mixture of 3 ml HNO_3_ and 7 ml H_2_SO_4_ was prepared.
- Control the highest digestion temperature at 220 °C
- Hold for 25 min

In this way, the tire samples were completely dissolved, the digestion solution was colourless and transparent, and a constant volume of 25 ml was also colourless and transparent.

The microwave digestion program can resist the interference of sample gram fluctuation. It is suitable for the pretreatment process of heavy metal detection of tire samples. The digestion process is characterized by safety, stability, high energy savings, and so on, which is suitable for general popularization.

However, there are some limitations to this study. As cars are a major contributor to traffic emissions, only car tires were selected as the test objects in this research. With the rise of electric vehicles, in the future, we can divide tires into cars, electric vehicles, Goods vehicles, and motorcycles for research purposes. In this paper, only the most representative Michelin tires were selected and only one microwave instrument was used. Thus, comparing different instruments and different brands is also a direction worth exploring. This study only focused on whether the digestion solution was complete from a qualitative analysis perspective. In the future, it could be considered from a perspective of quantitative analysis, for example, selecting a tire with a known heavy metals content and using different methods to dissolve it to see which is closer to the standard.

## Data Availability

All data generated or analyzed during this study are included in this published article.
